# Calculation of the Higher Order Dipole-Dipole Effect in Paramagnetic Crystals

**DOI:** 10.6028/jres.068A.010

**Published:** 1964-02-01

**Authors:** Paul H. E. Meijer

## Abstract

This report is an attempt to investigate the influence of dipole-dipole coupling in a paramagnetic spin system at low temperatures. It consists of two parts. The first part is a discussion of the use of *C_M_=C_H=_*_0_ for a system with mutual interaction. It is pointed out that only if the external field is large compared to the internal field is this equation correct.

The other part consists of a calculation of higher order correction of the dipole-dipole interaction on a system of paramagnetic spins which is subject to a crystalline field which we chose of the *Y*_2,0_ type. The total Hamiltonian consists of a spin Hamiltonian in accordance with this symmetry, a term representing the external magnetic field and the dipole-dipole interaction between the spins. The partition function is calculated by means of the Schwinger trace formula considering a representation in which the first two terms of the Hamiltonian are diagonal. The trace of the density matrix can be expressed as the trace of a product, one factor is the density matrix of the noninteracting spins, the other factor consists of a sum of commutators. These commutators are worked out in detail and the result is given in the form of a finite series over the quantum number *m.* There seems to be no obvious way to perform these summations.

## 1. Introduction

In a system of paramagnetic dipoles in a crystalline field the magnetic specific heat at constant magnetization is equal to the specific heat in zero magnetic field. This is easily seen from

$$C_{M}=C_{H}+T{\left(\frac{\delta M}{\delta T}\right)}_{H}{\left(\frac{\delta H}{\delta T}\right)}_{M}$$ (1.1)

and the fact that the magnetization in a system without any spatial correlation (i.e., ideal gas-like) is always proportional to *H* for small fields, hence *M* as well as (*dM/dT*) will go to zero if there is no field and we have *C_M_=C_H_*_=0_.

As a consequence of the lack of correlation *M*= 0 if *H*= 0, hence the relation should be *C_M=_*_0_*=C_H_*_=0_. The general question when *C_M_=C_H_*_=0_ is discussed in appendix 1.

If there is no crystalline field both these quantities are zero and the magnetic specific heat at constant *H* ≠ 0 is merely determined by the Curie law

$$C_{H}=\frac{CH^{2}}{T^{2}}$$ (1.2)

where *C* is the Curie constant.

For the case of dipole-dipole interaction between the spins the value of the specific heat at constant magnetic field was calculated by Van Vleck in 1937 [1].^1^ The result can be expressed in the limit of high temperatures in the form of an internal magnetic field *H_i_*, which has an average value zero, but a root mean square value *H_i_*, that is non-zero; then

$$C_{H}=\frac{C(H^{2}+\frac{1}{2}H_{i}^{2})}{T^{2}}.$$ (1.3)

We want to calculate *C_M_* again, the reason being that the ratio of *C_M_* to *C_H_* determines among other things the real part of the susceptibility at high frequencies. The result that *G_M_=G_H_*_=0_ was used in the calculation of this ratio [2], although this is correct only if a Curie law holds. Although the error is not very important, at least not at external fields large compared to the internal field, there is a certain inconsistency in the fact that *H_i_* ≠ 0 is in contradiction with the Curie law, which assumes the total absence of any mutual interaction.

In order to reconsider the equation of state, one can draw up a modification of the Curie equation by replacing the field *H* by either *H + H_i_* or by $\sqrt{H^{2}+H_{i}^{2}}$ and then recalculating the difference between *C_H_* and *C_M_* again; this is not equal to zero for *H* = 0. As a matter of fact it is infinitely large with the second choice

$$M=C\frac{H+H_{i}}{T}\to C_{H}-C_{M}=-\frac{C{\left(H+H_{i}\right)}^{2}}{T^{2}}$$ (1.4)

$$M=C\frac{{\left(H^{2}+H_{i}^{2}\right)}^{1/2}}{T}\to C_{H}-C_{M}=-\frac{C}{T^{2}}\frac{{\left(H^{2}+H_{i}^{2}\right)}^{3/2}}{H}.$$ (1.5)

The failure of such attempts is due to the fact that the Curie law is a high-temperature expansion in the case where the internal field is small. That is, the internal field itself is temperature dependent in such a way that it will vanish for *T*→∞. The difference between *C_M_* and *C_H_* is now

$$C_{H}-C_{M}=-\frac{CH^{2}}{T^{2}}+\frac{2H}{T^{2}}H_{i}\;\left(1+\frac{C}{T}\right)$$

since for high temperature *H_i_* is small and we find that this difference goes to zero for *H* = 0 and that the correction is proportional to *T*^−3^. This could have been obtained in a much simpler way if one assumed the Curie-Weiss law for the magnetization

$$M=C\frac{H}{T-\theta },$$ (1.6)

which leads to

$$C_{H}-C_{M}=-CH^{2}\frac{T}{{\left(T-\theta \right)}^{3}}.$$ (1.7)

The introduction of interaction between the spins is not in contradiction with the assumption that Δ*C* goes to zero for *H*→0.

The considerations above were mainly intended to provide a reasonable excuse to introduce an internal field by means of those terms in (*kT*)^−1^ which arise from the dipole-dipole interaction. These terms can be classified in two groups, classical and quantum mechanical. The quantum mechanical terms are the result of the noncommuting of the operators. For zero external field they occur only if there is a crystalline field splitting. It is the main purpose of this work to calculate these quantum mechanical effects in the internal field. The method followed is straightforward. The effects should be observable in the “intermediate” temperature region, that is, the high temperature expansion should hold far enough down, but not too close to the Curie temperature of the dipole-dipole interaction [3].

## 2. Operator Expansion

We will separate the Hamiltonian into two parts: the first part, ${ℋ}_{i}=a/k\;T$, contains the diagonal terms, the spin Hamiltonian in the crystalline and the external field; the second part, ${ℋ}_{2}=b/k\;T$ is the dipole-dipole interaction. It has no diagonal terms.

The expansion of the exponential needed for the evaluation of the partition function is

$$e^{-(a+b)}=e^{-a}+\sum_{n=1}^{\infty }{\left(-1\right)}^{n}e^{-a}S_{n}.$$ (2.1)

As is well-known [compare 4] *S_n_* contains the *b*-operator *n* times. If one actually wants to have concrete values for *S_n_* one has to perform another expansion, this time with respect to *a.* The practical question is how far do we calculate this double power series. The term *e*^−^*^a^*. *b* contained in *S*_1_ is a product of a purely diagonal term and a purely nondiagonal term. Hence the trace is zero [5], which means that the average value of the field created by the dipole-dipole interaction at a certain point is zero. The field has, however, a mean square value, and the first term in *S*_2_ is the major contribution to its value. This is a purely “classical” term in the sense that it is also nonzero for commuting operators. All first terms in *S_n_* are classical, but since we assume that the dipole-dipole interaction is small compared to the thermal energy of the system the description on the basis of the first term in *S*_2_ will be sufficient for the classical part as long as there is no evidence of an approaching cooperative phenomenon. Another argument for dropping the next terms is to notice that the range of the dipole-dipole interaction squared is *r*^−6^, and all higher classical terms with even *n*-value will give rise to weak interactions of extremely short range unless the temperature is so low that a strong correlation between the spins occurs.

The expressions for *S*_1_ and *S*_2_ are given by

$$S_{1}=\int_{0}^{1}ds_{1}b(s_{1});S_{2}=\int_{0}^{1}ds_{1}\int_{0}^{s_{1}}ds_{2}b(s_{1})b(s_{2})$$ (2.2)

and expanding *b* (*s*):

$$b(s)=e^{as}be^{-as}=b+s[a,b]+\ldots$$ (2.3)

The result of the integration is

$$\begin{array}{l} e^{-(a+b)}=e^{-a}\{1-S_{1}+S_{2}\}=e^{-a}(1-b+\frac{1}{2}[a,b]+\frac{1}{6}[a[a,b]]+\frac{1}{2}b^{2}\\ \;+\frac{1}{6}b[a,b]+\frac{1}{24}b[a[a,b]]+\frac{1}{3}[a,b]b+\frac{1}{8}{\left[a,b\right]}^{2}+\frac{1}{8}[a[a,b]]b. \end{array}$$ (2.4)

None of the terms of *S*_1_ contribute to the trace since they are the product of a number of diagonal matrices and *one* nondiagonal matrix.

The terms of *S*_2_ up to the second order in *b* are

$$\frac{1}{2}b^{2}+\frac{1}{6}b[a,b]+\frac{1}{3}[a,b]b.$$ (2.5)

It is shown in the app. 2 that the only contribution to the weighted trace is

$$\text{tr}\{e^{-a}(\frac{1}{2}\;b^{2}+\frac{1}{4}\;[b[a,b]])\}.$$ (2.6)

## 3. Calculation of the Double Commutator

In order to calculate the double commutator we introduce the following notation

$${ℋ}_{2}=g^{2}\beta^{2}\sum_{k\neq l}\frac{S_{k}S_{l}-3(S_{k}r_{kl})(S_{l}r_{kl})/r_{kl}^{2}}{r_{kl}^{3}}=\sum_{\alpha ,\beta }\sum_{k\neq l}S_{\alpha }^{k}\Theta_{\alpha \beta }^{kl}S_{\beta }^{l}.$$ (3.1)

Where the position indices are written as superscripts and the matrix θ is given by:

$$\begin{array}{ll} \Theta_{11}=g^{2}\beta^{2}(y^{2}+z^{2}-2x^{2})/r^{5}; & \Theta_{12}=\Theta_{21}=g^{2}\beta^{2}xy\\ \Theta_{22}=g^{2}\beta^{2}(x^{2}+z^{2}-2y^{2})/r^{5}; & \Theta_{23}=\Theta_{32}=g^{2}\beta^{2}yz\\ \Theta_{33}=g^{2}\beta^{2}(x^{2}+y^{2}-2z^{2})/r^{5}; & \Theta_{13}=\Theta_{31}=g^{2}\beta^{2}xz \end{array}$$ (3.1a)

leaving out all superscripts.

Since the leading part of the Hamiltonian is of the form (*a, b*, and *c* are constants)

$${ℋ}_{1}=\sum_{\text{positions}}aS_{z}^{2}+bS_{z}+c$$ (3.2)

we have to calculate the commutators *C_n_* and double commutators *D_n_*:

$$C_{n}=[S_{\beta },{\left(S_{z}\right)}^{n}]\;\text{and}\;D_{n}=[S_{\alpha },[S_{\beta },{\left(S_{z}\right)}^{n}]],$$ (3.3)

where all spins are at the same location. In order to avoid confusion with the superscripts, which have been omitted, the powers are placed outside parentheses. Table 1 shows that there are four nonzero values of *D*_1_. However two have zero’s in the diagonal, hence if multiplied by the (diagonal) weight factor only *α=β=x* or *y* will give a contribution. The same holds for *D*_2_. The trace of the two elements of *C* is *ħ*^2^*m.* The trace of the two elements of *D* is −*ħ*^3^ [3*m*^2^*−S*(*S*+1)]. This has to be inserted in the multiple sum over all positions containing two Θ’s and multipled by the weight factor exp $\left({ℋ}_{1}/k\;T\right)$.

Since both Hamiltonians are series over all positions and since all commutators with respect to two different positions are zero we have to figure out in which ways these two summations can “overlap”. The first commutator gives (*k*≠*l*):

$$[{ℋ}_{2},{ℋ}_{1}]=a\sum_{klm}\left[\sum_{\alpha \beta }S_{\alpha }^{k}\Theta_{\alpha \beta }^{kl}S_{\beta }^{l},(S_{z}^{m})\right]+b\sum_{klm}\left[\sum_{\alpha \beta }S_{\alpha }^{k}\Theta_{\alpha \beta }^{kl}S_{\beta }^{l},{\left(S_{z}^{m}\right)}^{2}\right].$$ (3.4)

The summation over *m* contains only two terms *m=k* and *m=l.* These terms are equal since the matrix Θ is symmetric in both the upper and lower indices and since *k≠l* the operators commute.

$$[{ℋ}_{2},{ℋ}_{1}]=2a\sum_{k\neq l}\sum_{\alpha ,\beta }\left[S_{\alpha }^{k},S_{z}^{k}\right]\Theta_{\alpha \beta }^{kl}S_{\beta }^{l}+2b\sum_{k\neq l}\sum_{\alpha \beta }\left[S_{\alpha }^{k},{\left(S_{z}^{k}\right)}^{2}\right]\Theta_{\alpha \beta }^{kl}S_{\beta }^{l}.$$ (3.5)

The double commutator introduces a sum over four positions, say *k, l*(*k*≠*l*) and *i, j*(*i*≠*j*). There are six different terms (table 2). The first four give all the same contribution because of the symmetry of Θ mentioned above, and so do the last two. Writing for short

$$f(S_{z}^{m})=a{\left(S_{z}^{m}\right)}^{2}+b(S_{z})$$

we have

$$\begin{array}{l} {}[[{ℋ}_{2},[{ℋ}_{2},{ℋ}_{1}]]=\sum_{\underset{\alpha^{\prime }\beta^{\prime }}{\alpha \beta }}(8\sum_{\;\begin{array}{l} jkl\\ \text{all different} \end{array}}\{[S_{\alpha }^{k},[S_{\alpha^{\prime }}^{k},f(S_{z}^{k})]]\Theta_{\alpha \beta }^{kj}\Theta_{\alpha^{\prime }\beta^{\prime }}^{kl}S_{\beta }^{j}S_{\beta^{\prime }}^{l}\\ \;+[S_{\alpha }^{k},S_{\alpha^{\prime }}^{k}]\Theta_{\alpha \beta }^{kj}\Theta_{\alpha^{\prime }\beta^{\prime }}^{kl}[S_{\beta^{\prime }}^{l},f(S_{z}^{l})]S_{\beta }^{j}\}+4\sum_{k\neq l}\{S_{\alpha^{\prime }}^{k}[S_{\alpha }^{k},f(S^{k})]\Theta_{\alpha \beta }^{kl}\Theta_{\alpha^{\prime }\beta^{\prime }}^{kl}[S_{\beta^{\prime }}^{l},S_{\beta }^{l}]\\ \;+[S_{\alpha^{\prime }}^{k},[S_{\alpha }^{k},f(S^{k})]]\Theta_{\alpha \beta }^{kl}\Theta_{\alpha^{\prime }\beta^{\prime }\;}^{kl}S_{\beta^{\prime }}^{l}S_{\beta }^{l}-[S_{\alpha^{\prime }}^{k},[S_{\alpha }^{k},f(S^{k})]]\Theta_{\alpha \beta }^{kl}\Theta_{\alpha^{\prime }\beta^{\prime }}^{kl}[S_{\beta^{\prime }}^{l},S_{\beta }^{k}]\}), \end{array}$$ (3.6)

using for the second set of terms the following commutator identity:

[AB,CD]=AC[BD]+[AC]BD−[AC][BD]

if only

[AC]≠0and[BD]≠0

and all other commutators are zero. Only the diagonal part of this double commutator contributes to the partition sum, and as a result of this the summation over *α, β*, *α*′ and *β*′ has a number of restrictions:

First term: *α* = *α*′ = *x* or *y; β* = *β*′ = *z.*
Second term is always zero since [*S_β_, f*(*S*_2_)] has no diagonal elements for every *β*′.
Third term: *α = α*′ = *x* or *y*, and *β, β*′ = *x*, *y* or *y, x.*
Fourth term: *α* = *α*′ = *x* or *y* and *β*′, *β* = *z*. In case it is *x, y* it will be canceled by the diagonal contribution from *y*, *x.*
Fifth term: *α* = *α*′ = *x* or *y* and *β*′, *β* = *x, y* or *y, x.*

The diagonal parts of the spin operators to the second power are:

$$\begin{array}{c} {\left(S_{x}^{2}\right)}_{D}={\left(S_{y}^{2}\right)}_{D}=\frac{1}{2}[S(S+1)-S_{z}^{2}]\\ {\left(S_{x}S_{y}\right)}_{D}=-{\left(S_{y}S_{x}\right)}_{D}=\frac{\hbar S_{z}}{2i} \end{array}$$

and using table 1 we find (combining the first and fourth term):

$$\begin{array}{l} {\left[{ℋ}_{2},[{ℋ}_{2},{ℋ}_{1}]\right]}_{\text{Dlag}.}=8\sum_{\underset{k\neq l}{k\neq j}}{\left(i\hbar \right)}^{2}\{\frac{a}{2}\left[3{\left(S_{z}^{k}\right)}^{2}-{\left(S^{k}\right)}^{2}\right]\left(\Theta_{xz}^{kj}\Theta_{xz}^{kl}-\Theta_{yz}^{kj}\Theta_{yz}^{kl}\right)S_{z}^{j}S_{z}^{l}+\\ \;+bS_{z}^{k}\left(\Theta_{xz}^{kj}\Theta_{xz}^{kl}+\Theta_{yz}^{kj}\Theta_{yz}^{kl}\right)S_{z}^{j}S_{z}^{l}\}+\sum_{\underset{\alpha^{\prime }\beta^{\prime }}{\alpha \beta }}4\sum_{k\neq l}\left(\left[S_{\alpha }^{k},f(S^{k})]S_{\alpha^{\prime }}^{k}\Theta_{\alpha \beta }^{kl}\Theta_{\alpha^{\prime }\beta^{\prime }}^{kl}[S_{\beta^{\prime }}^{l},S_{\beta }^{l}\right]\right). \end{array}$$ (3.7)

The last term is calculated with the help of

$$[S^{\pm },S_{z}^{2}]S^{\mp }=(\mp 2\hbar S_{z}+\hbar^{2})S^{\pm }S^{\mp }+\hbar^{2}(S^{2}-3S_{z}^{2})\pm \hbar^{3}S_{z}\mp 2\hbar S_{z}(S^{2}-S_{z}^{2}).$$ (3.8)

The summation over *α*, *β*, *α′, β′* contains 8 terms. It turns out, however, that 4 cancel mutually: if *α* and *β* are equal (both *x* or both *y*) the subscripts *α′β′* are either *x* and *y* or *y* and *x.* Both have the same coefficient $\Theta_{xx}^{kl}\Theta_{xy}^{kl}$ but the commutators have the opposite sign. The fact that these cancel could have been seen on the following general grounds. A commutator is anti-Hermitian and a double commutator is Hermitian. Hence the last factor has imaginary diagonal elements and the total expression has real diagonal elements, and the first factor must have an imaginary diagonal part:

$$\begin{array}{l} {}[S_{y},\;f{\left(S_{z}\right)}^{2}]S_{x}=-[S_{x},\;f(S_{z})]S_{y}\\ \;=\frac{i}{4}([S^{+},\;f(S_{z})]S^{-}-[S^{-},\;f(S_{z})]S^{+}\\ \;=-\frac{i}{2}[a\{2\hbar S_{z}(S^{2}-S_{z}^{2})-\hbar^{3}S_{z}\}+b\hbar (S^{2}-S_{z}^{2})]. \end{array}$$ (3.9)

The final result for the last term is:

$$4\sum_{k\neq l}\hbar^{2}[(\Theta_{xx}^{kl}\Theta_{yy}^{kl})-{\left(\Theta_{xy}^{kl}\right)}^{2}][2aS_{z}^{2}(S^{2}-S_{z}^{2}-\frac{1}{2}\hbar^{2})+bS_{z}(S^{2}-S_{z}^{2})].$$ (3.10)

In order to obtain a value for the partition functions the trace has to be calculated numerically since the sums of the type

$$\sum_{p}=\sum_{m=-S}^{S}m^{p}e^{am^{2}+bm}\;(p=0,1,2,3)$$ (3.11)

cannot be evaluated in closed form.

## 4. Conclusion

The complication of the calculation lies mainly in the fact that the commutator of two spin operators is not a *c*-number but again an operator. This makes many tricks used to evaluate exponential operators useless. For instance, one could have written the original total Hamiltonian with step up and step down operators *S*^+^ and *S*^−^. The expansion of the exponential would have created a polynomial containing *S*^+^, *S*^−^ and *S_z_* to certain powers. A general conclusion is that only those terms which contain an equal number of *S*^+^ and *S*^−^ at the same location will have a nonzero diagonal element. To calculate the actual value, however, one has to shift the *S*^+^ and *S*^−^ operator such that they are all at the end or the beginning of the monomial and in conjunction to each other. This rearrangement of operations is accomplished with the help of Wick’s theorem if we deal with creation and annihilation operations. It is still possible to formulate a Wick-like theorem for spin one-half. This was done by Kenan [6]. It introduces an additional numerical factor into each diagram. For spin not equal to one-half, his treatment fails. The crucial point is that only for spin one-half is (*S_z_*)^2^ a *c*-number. Otherwise *S_z_* is an operator and hence can not be taken outside the trace and the contraction operator cannot be defined. These considerations indicate that it seems unavoidable to calculate the trace straightforwardly. Actually the straightforward calculation consists of doing the same steps as in the Wick-theorem.

Finally, the summation of the positions has to be performed. It is possible to restrict this to the immediate environment since the radial dependence decreases very rapidly.

## Figures and Tables

**Table 1:** 

*D*_1_/(*iħ*)^2^			
	*x*	*y*	*z*
			
*x*	−*S_z_*	0	0
*y*	0	−*S_z_*	0
*z*	*S_x_*	*S_y_*	0

	*D*_2_/(*iħ*)^2^		
	*β*=*x*	*y*	*z*
			
*α*=*x*	$2(S_{z}^{2}-S_{y}^{2})$	−(*S_y_S_x_*+*S_x_S_y_*)	0
*y*	(*S_x_S_y_*+*S_y_S_x_*)	$2(S_{x}^{2}-S_{z}^{2})$	0
*z*	−(*S_z_S_x_*+*S_x_S_z_*)	(*S_z_S_y_+S_y_S_z_*)	0

**Table 2:** 

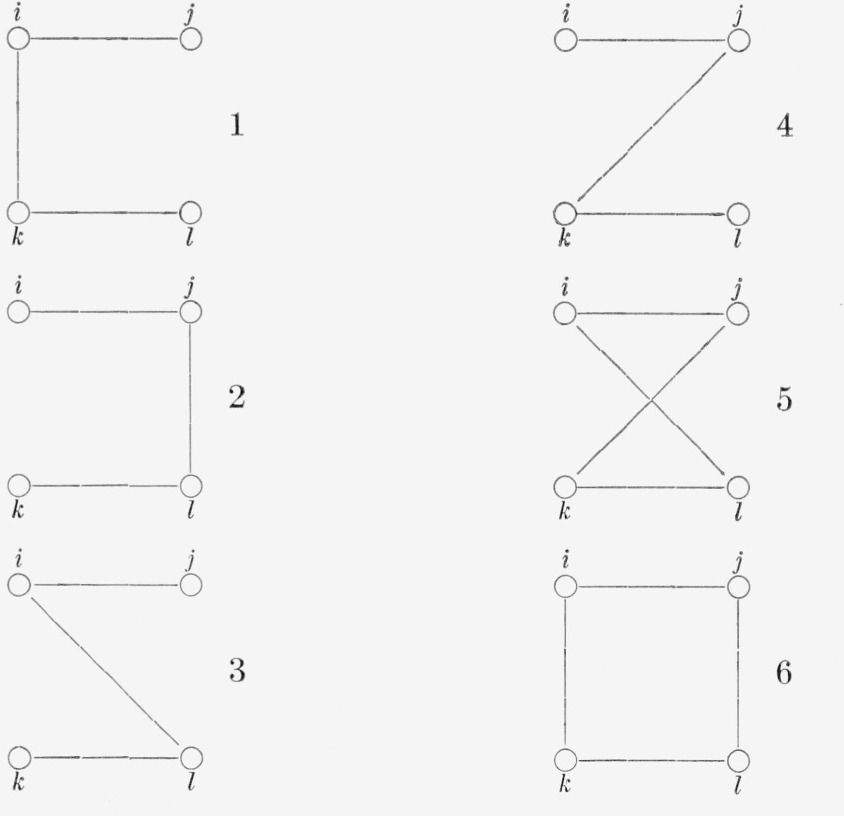

## Footnotes

1 Figures in brackets indicate the literature references on page 118.

## 6. Appendix 1

We want to investigate under what conditions *C_M_*=*C_H_*_=0_ for arbitrary *H.* To calculate the specific heat at constant *H* we find from the Maxwell relation *∂S/∂H* = *∂M/∂T*

$$C_{H}=T\int_{0}^{H}\frac{\partial^{2}M}{\partial T^{2}}dH+C_{H=0}$$

the second term at the right-hand side of (11) is

$$C_{M}=C_{H}+T{\left(\frac{\partial M}{\partial T}\right)}^{2}/\frac{\partial M}{\partial H}$$

and the condition *C_M_*=*C_H_*_=0_ gives

$$T\int_{0}^{H}\frac{\partial^{2}M}{\partial T^{2}}dH=T{\left(\frac{\partial M}{\partial T}\right)}^{2}/\frac{\partial M}{\partial H}$$

which leads to the differential equation

$${\left(\frac{\partial M}{\partial H}\right)}^{2}\frac{\partial^{2}M}{\partial T^{2}}-2\frac{\partial^{2}M}{\partial T\partial H}\frac{\partial M}{\partial T}\frac{\partial M}{\partial H}+{\left(\frac{\partial M}{\partial T}\right)}^{2}\frac{\partial^{2}M}{\partial H^{2}}=0$$

which is fulfilled by

$$M=f\left(\frac{H+c_{1}}{T+c_{2}}\right)-f\left(\frac{C_{1}}{C_{2}}\right)$$

where *f* is an arbitrary function and *c*_1_ and *c*_2_ two constants. The initial condition forces us to take *c*_1_ = 0. For *kT* larger than the crystal field splitting this condition is fulfilled.

## 7. Appendix 2

From the sum and difference

$$b[a,\;b]+[a,\;b]b=ab^{2}-b^{2}a\;b[a,\;b]-[a,\;b]b=[b,\;[a,b]]$$

we find

$$b[a,\;b]=\frac{1}{2}[b,[a,\;b]]+\frac{1}{2}ab^{2}-\frac{1}{2}b^{2}a\;[a,\;b]b=\frac{1}{2}[b,[a,\;b]]-\frac{1}{2}ab^{2}+\frac{1}{2}b^{2}a.$$

Hence the relevant terms in (2.5) are

$$\frac{1}{6}b[a,\;b]+\frac{1}{3}[a,\;b]b=\frac{1}{12}[b,[a,\;b]]+\frac{1}{12}ab^{2}-\frac{1}{12}b^{2}a+\frac{1}{6}[b,[a,\;b]]-\frac{1}{6}ab^{2}+\frac{1}{6}b^{2}a=\frac{1}{4}[b,[a,\;b]]-\frac{1}{12}ab^{2}+\frac{1}{12}b^{2}a.$$

Since *a* is diagonal we have

$$\text{tr}.\{(f(a)ab^{2})\}=\text{tr}.\{(f(a)b^{2}a)\},$$

hence the contribution to the trace is

$$\text{tr}.\{f(a)\frac{1}{4}[b[a,b]]\}.$$
